# Bond strength of adhesive resin cement with different adhesive systems

**DOI:** 10.4317/jced.53099

**Published:** 2017-01-01

**Authors:** Fabrizio Lorenzoni e Silva, Saulo Pamato, Milton-Carlos Kuga, Marcus-Vinicius-Reis Só, Jefferson-Ricardo Pereira

**Affiliations:** 1 DDS, MSc, Program of Health Science, University of Southern Santa Catarina, Tubarão, Santa Catarina, Brazil; 2 DDS, PhD, Department of Restorative Dentistry, Araraquara Dental School, Araraquara, SP, Brazil; 3 DDS, MSc, PhD, Departament of Endodontics, Federal University of Rio Grande do Sul

## Abstract

**Background:**

To assess the immediate bond strength of a dual-cure adhesive resin cement to the hybridized dentin with different bonding systems.

**Material and Methods:**

Fifty-six healthy human molars were randomly divided into 7 groups (n=8). After 3 longitudinal sections, the central cuts were included in PVC matrix and were submitted to dentin hybridization according to the groups: G1 - etch & rinse system with 3-step (Apder™ Scotchbond™ Multi-Purpose, 3M ESPE), G2 - etch & rinse system with 3-step (Optibond™ FL, Kerr), G3 - etch & rinse system with 3-step (All-Bond 3®, Bisco), G4 - etch & rinse simplified system (Adper™ Single Bond 2, 3M ESPE), G5 - self-etching system with one step (Bond Force, Tokuyama), G6 - universal system in moist dentin (Single Bond Universal, 3M ESPE), G7 - universal system in dry dentin (Single Bond Universal, 3M ESPE). Then all groups received the cementing of a self-adhesive resin cement cylinder (Duo-link, Bisco) made from a polypropylene matrix. In the evaluation of bond strength, the samples were subjected to the microshear test and evaluated according to the fracture pattern by optical microscopy.

**Results:**

The Kruskal-Wallis test suggests a statistically significant difference between groups (*p*=0,039), and Tukey for multiple comparisons, indicating a statistically significant difference between G3 and G4 (*p*<0.05). It was verified high prevalence of adhesive failures, followed by mixed failure and cohesive in dentin.

**Conclusions:**

The technique and the system used to dentin hybridization are able to affect the immediate bond strength of resin cement dual adhesive.

** Key words:**Adhesion, adhesive resin cement, adhesive systems, microshear.

## Introduction

The increase in the demand for satisfactory and biomechanically aesthetic restorations, whether direct or indirect, has led to research and development of materials that naturally reproduce shape, color, texture, and function of the lost tooth structure. The success of indirect restorative techniques depends on cementing agents that will bond the restorations to the remaining tooth structure (1-3).

Resin cements depend on the pretreatment of the enamel and/or dentin surface, except for the self-adhesive ones (2-4). The role of adhesive systems is to create bonding mechanisms between the restorative material, the resin cement, and the substrate of the tooth surface. These mechanisms require surface etching (enamel and/or dentin), application of a hydrophilic primer that increases surface wettability and adhesive component, which is a fluid resin that binds the conditioned tooth surface to direct or indirect restorations (3,5).

The form and bond strength of adhesive systems on dentin and enamel behave differently. Because of this behavior, numerous studies have been conducted over time, which resulted in different materials and techniques for the conditioning and treatment of each surface (2,3). Generations of adhesive systems have been created, now converging to preconditioning systems, which require acid etching prior to the application of the adhesive system, and self-conditioning systems that do not require previous acid application as they have acidic components in their formula (2-5).

Given the many techniques and materials available in the market, it is necessary to perform experimental studies aimed at a better understanding of their effectiveness, as well as to enable the creation of clearer protocols for each clinical indication. The hypothesis of this study stated that there would be significant differences between the adhesive systems tested.

## Material and Methods

This study was approved by the Research Ethics Committee of the University of Southern Santa Catarina (UNISUL). Fifty-six freshly extracted caries-free human molars with similar dimensions and anatomic structure were used in this study.

The teeth were cleaned from remnant soft tissue and stored in 0.5% chloramine T at room temperature during the first 7 days after extraction, and thereafter stored in distilled water at 5˚C for a maximum of 6 months.

Three standard longitudinal sections were performed for each tooth, dividing them into four by using diamond discs at low speeds coupled to a standard machine (ISOMET® 1000, Buehler, Lake Buff, IL, USA). The two central portions of each specimen were embedded in a rigid, ring-shaped PVC matrix (Tiger, Joinville, Brazil) across sections of tubes with a diameter of 5 cm, filled with acrylic resin (JET, Classic, São Paulo, SP, Brazil). The set was smoothed and polished in a specific machine (Arotec® APL-4, São Paulo, SP, Brazil) using silicon carbide sandpaper sheets in descending order of grain (#200, #400, #600, and #1200) and an aluminum oxide-based polishing paste (Diamond R, FGM, Joinville, Brazil). The specimens were divided into seven groups (n=8), according to the type of adhesive system used: G1 - total acid etching with 37% phosphoric acid (Dentsply, Konstanz, Germany), associated with a three-step adhesive system (Adper™ Scotchbond™ Multi-Purpose, 3M ESPE, St. Paul, MN, USA); G2 - total acid etching with 37% phosphoric acid (Dentisply®, Konstanz, Germany), associated with a three-step adhesive system (OptiBond™ FL, Kerr, Orange, CA, USA); G3 – total acid etching associated with a three-step system (All-Bond 3®, Bisco, Schaumburg, IL, USA); G4 - total acid etching with 37% phosphoric acid (Dentisply®, Konstanz, Germany), associated with a simplified two-step system (Adper™ Single Bond 2, 3M ESPE, St. Paul, MN, USA); G5 – one-step self-etching system (Bond Force, Tokuyama, Osaka, Japan); G6 – total acid etching with 37% phosphoric acid (Dentsply, Konstanz, Germany), associated with a universal adhesive system (Single Bond Universal, 3M ESPE, St. Paul, MN, USA) in a moist dentin environment; G7 – total acid etching with 37% phosphoric acid (Dentsply, Konstanz, Germany), associated with a universal adhesive system (Single Bond Universal, 3M ESPE, St. Paul, MN, USA) in dry dentin. All surfaces were then light-cured for 20 seconds with a light-curing 1400-watt lamp (Valo®, Ultradent, South Jordan, UT, USA) at a distance of 0.5 cm.

After the surface treatment of the specimens, a cylindrical increment of a dual-cure resinous cement (Duo-Link, Bisco, Schaum-burg, IL, USA) was made perpendicularly to the hybridized dentin using a lentulo-type drill. The cement was then homogenized inside the orifice of a polypropylene matrix (Bonding Mold Inserts, Ultradent, South Jordan, UT, USA) and subsequently placed on a metal device (BondingClamp, Ultradent, South Jordan, UT, USA). This set was kept in contact with the specimen so that the inserted resin cement came into direct contact with the treated dentin. Then, it was photoactivated for 40 seconds by using a LED curing light (Valo®, Ultradent, South Jordan, UT, USA). The matrix/metal device assembly was removed after complete polymerization of the increment.

Aligned to a universal tensile testing machine (EMIC DL2000, São José dos Pinhais, PR, Brazil), the specimens were placed in a metal device and the cylinder was tied with a steel wire 0.2 mm in diameter, parallel to the exposed tooth surface. Then, a shear force of 0.5 mm/min was applied until a fracture occurred. At the end of the test, the values were recorded in Newton (N) by using the Tesc 3.04 software (EMIC, São José dos Pinhais, PR, Brazil) and were converted to MegaPascal (MPa) following the formula: MPa=Newton/area (mm2).

All specimens submitted to microshear test were prepared for the standard analysis of fracture under a stereomicroscope (Stemi DV4, Zeiss Universal Microscope, Jena, Germany) and optical microscope (N107, Coleman, Santo André, SP, Brazil) at 40X magnification. The fracture patterns were classified as follows: (1) adhesive fracture: rupture at the bond interface; (2) dentin cohesive failure: complete rupture of dentin; (3) cement cohesive fracture: complete rupture of the cement cylinder; (4) Mixed fracture: rupture encompassing dentin and resin cylinder.

Analyses of Variance (ANOVA) and Tukey test for multiple comparisons were used to examine statistically significant differences between groups. The significance level was set at 0.05.

## Results

The Shapiro-Wilk test was applied to test normal distribution of data, pointing to the need for a non-parametric test (*p*<0.05) to determine statistically significant differences between groups. The Kruskal-Wallis test (*p*=0.05) was used because there was only one factor (dentin substrate) to be analyzed.

The results of the Kruskal-Wallis test identified statistically significant differences between the groups tested (*p* = 0.039). The data were analyzed using the Tukey test for multiple comparisons ( Table 1).

**Table 1:**
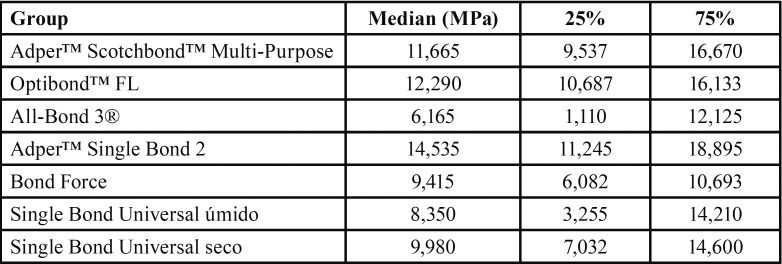
Type of fractures per group.

As for the analysis of the type of fracture, the data obtained by optical microscopy were displayed by group in  Table 2.

**Table 2:**
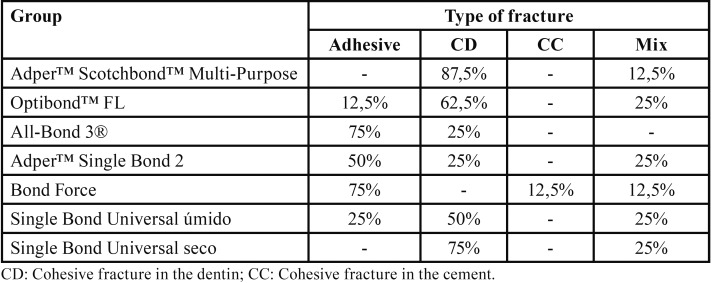
Groups, median, and upper and lower quartile values.

## Discussion

Based on the results of this study, dentin hybridization with different bonding systems showed distinct adhesion performances according to the system and technique used. Whereas most groups showed no statistically significant difference, the specimens hybridized with Adper™ Single Bond 2 (3M ESPE) showed higher scores of microshear bond strength. Therefore, the null hypothesis stating that the dentin hybridization system and technique will not affect immediate bond strength of the adhesive resin cement was rejected.

Conventional bonding systems are widely considered important in dentistry and have shown long tradition of good results in clinical and laboratory assessments (5). However, score discrepancies presented by those systems in the present study reinforce the argument that adhesives with high bond strength should not be the sole criterion for successful bonding (6). Currently, the superior quality of the adhesive interface and the ideal relationship between thickness and bond strength has been claimed as the main reasons for its excellent clinical performance in face of the contemporary self-etching systems (7).

This concept is in agreement with the experiment conducted by Aw et al. (8) in comparing the clinical behavior of two conventional systems over three years. The authors found very similar bond strength patterns between the two-step and three-step systems. Loguercio *et al.* (9) have claimed that bond strength of simplified systems can reach scores 51-100%, whereas Wilder *et al.* (10) have reported cores between 69-100% for the current three-step systems. Nevertheless, it is important to note that such understanding is not unanimous in the literature (11). In publishing a systematic review of clinical studies, Peumans *et al.* (12) have claimed that such discrepancy is due to technical sensitivity, especially because of the presence of polyalkenoic acid in the composition of the simplified systems. This acid is present in almost all bonding systems manufactured by 3M ESPE, and assigns a high molecular weight to the formula. This compound can hamper resin monomer interdiffusion in the demineralized collagen network, promoting the formation of areas in which fibrils are not permeated by resinous compounds. From another perspective, this compound is capable of forming complexes in the conditioned substrate, which can stabilize the bonding interface by stress dissipation. However, under laboratory conditions, comparison between bond strength values provided by different adhesive systems should be viewed with caution, given that many factors can influence the results, such as the type of test applied, the tested area, and stress distribution that occurs in each assay (13).

Contrastingly, analysis of the simplified adhesive system revealed different results from those described by De Munck *et al.* (14) Whereas the authors of that study claimed that the high hydrophobicity of the formula components can prevent adequate penetration of the adhesive monomers in the collagen matrix, thus compromising hybrid layer quality, the present study identified a superior immediate bond strength of the Adper™ Single Bond 2 (3M ESPE) system, although not differing from most of the other systems used.

Moreover, result disparities presented by the conventional three-step systems confirm the findings published Pashley *et al.* (15) According to authors, the presence of ethanol as solvent in the formulation of the primer is capable of promoting a chemical dehydration of the demineralized collagen matrix, resulting in a lateral shrinkage of collagen fibers, an increase in the interfibrillar spacing width, and therefore, a reduction in collagen matrix hydrophilicity.

In order to eliminate the technical sensitivity characteristic of conventional bonding systems, a self-etch protocol was proposed in the early 1990s (16). These systems differ from the conventional ones by the absence of the substrate rinse step, thus reducing the clinical time. Furthermore, combining a surface demineralization protocol to the permanence of the smear layer, often results in less postoperative sensitivity, as well as lower bond strength. However, it is known that quality of the bonding interface is closely related with the extent of infiltration of the resin monomers into the collagen matrix previously demineralized (17). Considering the limitations of the *in vitro* findings, the results of this study are consistent with the principles set out in other published works, which have associated self-etching systems to lower bond strength scores (18).

Analysis of the failure modes exhibited higher prevalence of dentin cohesive fracture (46.43%), followed by adhesive failure (33.93%) and mixed failure (17.86%). These results can be explained by the maximum penetration of resin monomers guaranteed by the previous use of a conditioning agent. On the other hand, these results corroborate the argument made by Oilo and Austrheim (19). According to these authors, conventional studies using shear strength methodology will most often present dentin cohesive failures, being unable to reproduce in a reliable way the adhesive strength of the bonding agents.

Considering the limitations of this *in vitro* study, the dentin hybridization can affect the immediate bond strength of dual-cured resin cements. Among the protocols adopted in this study, Adper™ Single Bond 2 system presented the highest bonding scores, being the only group to show a statistically significant difference when compared to the All-Bond 3® system.
